# Social dilemma in the excess use of antimicrobials incurring antimicrobial resistance

**DOI:** 10.1038/s41598-022-25632-1

**Published:** 2022-12-06

**Authors:** Hiromu Ito, Takayuki Wada, Genki Ichinose, Jun Tanimoto, Jin Yoshimura, Taro Yamamoto, Satoru Morita

**Affiliations:** 1 Department of International Health and Medical Anthropology, Institute of Tropical Medicine, Nagasaki University, Nagasaki, Japan; 2 Graduate School of Human Life and Ecology, Osaka Metropolitan University, Osaka, Japan; 3 Graduate School of Science and Technology and Department of Mathematical and Systems Engineering, Shizuoka University, Shizuoka, Japan; 4 Department of Energy and Environmental Engineering, Interdisciplinary Graduate School of Engineering Sciences, Kyushu University, Fukuoka, Japan; 5 Department of Advanced Environmental Science and Engineering, Faculty of Engineering Sciences, Kyushu University, Fukuoka, Japan; 6 Marine Biosystems Research Center, Chiba University, Chiba, Japan; 7 Department of Biological Science, Tokyo Metropolitan University, Tokyo, Japan; 8 University Museum, The University of Tokyo, Tokyo, Japan

**Keywords:** Infectious diseases, Evolution, Epidemiology, Psychology and behaviour, Public health

## Abstract

The emergence of antimicrobial resistance (AMR) caused by the excess use of antimicrobials has come to be recognized as a global threat to public health. There is a ‘tragedy of the commons’ type social dilemma behind this excess use of antimicrobials, which should be recognized by all stakeholders. To address this global threat, we thus surveyed eight countries/areas to determine whether people recognize this dilemma and showed that although more than half of the population pays little, if any, attention to it, almost 20% recognize this social dilemma, and 15–30% of those have a positive attitude toward solving that dilemma. We suspect that increasing individual awareness of this social dilemma contributes to decreasing the frequency of AMR emergencies.

## Introduction

Antimicrobials have saved the lives of many people facing various microbial infections, such as bacterial, viral, and fungal infections, since the discovery of the first antibiotic, penicillin, 1928. The excess use of antimicrobials has led to evolution toward antimicrobial resistance (AMR). AMR is currently frequently seen under the excess use of antimicrobials. Drug resistance is frequently acquired because the microorganisms inhabiting patients face strong selection pressure from antimicrobials. The emergence of AMR is a serious threat to human health. In 2013, it was reported that the number of deaths due to AMR reached 700,000 worldwide, and it is expected to increase to 10 million by 2050^1^. The number of deaths associated with bacterial AMR in 2019 was estimated to be 4.95 million, including 1.27 million deaths strictly attributable to bacterial AMR^2^. Faced with such circumstances, the World Health Organization (WHO) and health authorities in many countries continuously warn against the excessive use of antimicrobials^3,4^. However, it is estimated that approximately half of antimicrobial prescriptions for acute medical patients in hospitals are not appropriate^5^. Recently, antibiotics were prescribed to most (72%) patients hospitalized for pneumonia due to COVID-19, but only 8% of patients had bacterial pneumonia for which antibiotics should be administered^6^. Because of the current COVID-19 pandemic, the preventive use of antimicrobials has become increasingly prevalent, resulting in an increased risk of AMR. The spread of AMR not only causes an increase in the number of fatal infections in vulnerable people with compromised immunity who are unlikely to experience spontaneous recovery but also undermines current medical procedures, such as surgery and organ transplantation, that depend on the effect of antimicrobials^1–7^. The worldwide spread of AMR is a major medical threat to modern society, and it is urgent that we seek and design containment measures not only at the individual level but also at a social level^7^.

**Figure 1:**
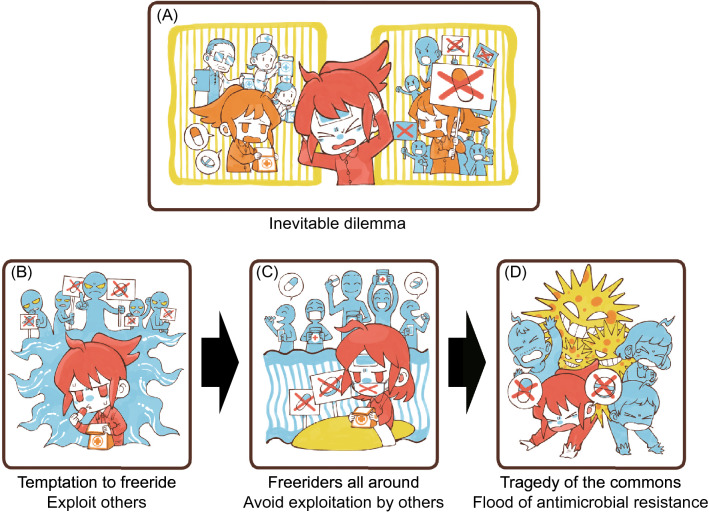
The social dilemma in the excess use of antimicrobials and the resulting emergence/spread of antimicrobial resistance (AMR). (**A**) If everyone tries to free ride (use antimicrobials freely), a flood of AMR is inevitable. Do you (and everybody) use antimicrobials, ignoring AMR or do you not use antimicrobials unless necessary, to avoid AMR? (**B**) If you free ride on the cooperative behavior of others, only you will receive benefit from the antimicrobials. People have a common understanding (public stance) to refrain from free use of antimicrobials. However, they will also be tempted to use antimicrobials themselves secretly. The rational choice is to free ride (use antimicrobials), increasing the risk of AMR. Do you? (**C**) As more people use antimicrobials unnecessarily, the incentive for cooperators (you) to refrain from using antimicrobials decreases. The rational choice is to use antimicrobials (free ride) because everybody uses the drugs. Do you? (**D**) The tragedy of the commons appears: the risk of AMR flooding is increasing. A flood of AMR is an undesirable outcome for free riders. Here, effective antimicrobials are common and can be exploited by free riders.

If all people use antimicrobials as little as possible, the risk of AMR emergence can be minimized. From an individual perspective, however, every person wants to use common antimicrobials to prevent possible infections even if the diagnosis of infections is suspicious (Fig. 1A). Prophylactic use of antimicrobials should be considered for individuals who have a risk of such infections. In contrast, from the social perspective, such antimicrobial use is known to cause AMR at a low rate, especially in hospitals. Such conflict has been noted as the social dilemma behind AMR by policy makers, hospital administrators, and medical prescribers^7–12^. This dilemma has been analyzed as an antimicrobial prescription game among physicians (players)^10,12^. These are important previous studies that envision a medically ‘provider-driven’ society in which antimicrobial prescriptions are determined independently of the patient’s consent. However, informed consent is recently established in some countries, patients can decide their own treatments. In such a ‘patient-driven’ society, the social dilemma can only be resolved through the efforts not only of the medical system but also of actions to all citizens who could become patients. Therefore, this dilemma is also considered a game among all patients (public), as noted above from the individual perspective. As healthcare, which was previously provider-driven, is now gradually shifting to be patient-driven, this dilemma should be considered a game among patients.

There is no overall best strategy unifying individual and societal perspectives (Fig. 1). This is an example of ‘the tragedy of the commons,’ where depletion occurs due to uncontrolled consumption of common resources (common goods)^13^. Here, the common resource is the ‘repertoire of effective antimicrobials’ or ‘public health’ itself that would be lost (consumed) by emergence of AMR. In game theory, a self-optimizer exploiting those who avoid the excess use of antimicrobials for societal benefit is a ‘free rider’. On the other hand, we can consider a player who refrains from the casual use of antimicrobials as a ‘cooperator’ in the context of evolutionary game theory. This is because cooperators will take antimicrobials as little as possible, thus, cooperators are contributing to preventing AMR emergence/spreading. In this sense, cooperators are evolutionarily disadvantaged because they are not adopting a self-optimizing strategy that minimizes the risk and burden to their health. In this situation, the number of free riders becomes a key factor determining optimality. First, when free riders are very few, because the risk of AMR is minimum (negligible), all patients favor using antimicrobials, resulting in an increase in free riders (users) (Fig. 1B). When antimicrobials become commonly used, the AMR risk becomes prevailing, patients grow incentives to avoid them to lower the AMR risk. However, when free riders increase in numbers, a patient is prompted to use antimicrobials because the risk of AMR has already increased by other free riders, thus, the benefit of the antimicrobials outweighs the probability of AMR for that patient (Fig. 1C). Interestingly, the current social dilemma may promote the use of antimicrobials against decision makers both when there are many free riders and when there are few. Note that the free riders themselves (Fig. 1B) and individuals (players) surrounded by many free riders (Fig. 1C) have different motivations for using antimicrobials. The former (Fig. 1B) uses antimicrobials to exploit the cooperative behavior of others, because they believe that antimicrobials will save their lives, which is a purely self-centered perspective devoid of any consideration of what others are doing. Meanwhile the latter (Fig. 1C) uses antimicrobials to prevent own risk of infection but to ignore the already unavoidable risk of AMR (i.e., to prevent exploitation by the non-cooperative behavior of others in game-theoretic interpretation)^14^. When the number of antimicrobial users further increases, the number of deaths related to AMR increases (Fig. 1D). How is this social dilemma recognized by the people in society? How seriously is this social dilemma currently viewed by society? To solve this problem of AMR, as a first step, we surveyed the individual attitudes toward this AMR social dilemma in several countries/areas.

We designed a questionnaire to observe a social dilemma by placing two types of imaginary artificial-intelligence (AI) physicians who perform medical practice from either an individual or societal perspective, following autonomous vehicle AI studies^15,16^. This setting allows us to ask respondents whether they recognize the social dilemma without a direct question, including the explanation of this dilemma (for details, see Supplementary Information).

## Results

A web survey entitled “Survey on Medical Advancement” was administered to 41,978 people in 8 countries/areas: Japan (2 panels pre- and post-COVID-19), the United States, the United Kingdom, Sweden, Taiwan, Australia, Brazil, and Russia (Table S1). All respondents were classified into four categories based on their AI preferences: (i) *Democratic cooperator* (abbreviated by *DC*): Choose World-AI for both their own and strangers’ diagnoses; (ii) *Shrewd egotist* (abbr. by *SE*): Choose World-AI for strangers’ diagnosis but Individual-AI for their own; (iii) *Altruistic masochist* (abbr. by *AM*): Choose the World-AI for their own but the Individual-AI for strangers’; and (iv) *Indifferent egotist* (abbr. by *IE*): Choose the Individual-AI for both their own and strangers’ diagnosis. Here, *SE* expresses the social dilemma. *DCs* are the most open to controlling AMR, *SEs* recognize the importance of AMR prevention, and *IEs* ignore or understate the AMR problem. *AMs* are hard on themselves but caring toward others, and few or no *AMs* are expected.

**Figure 2:**
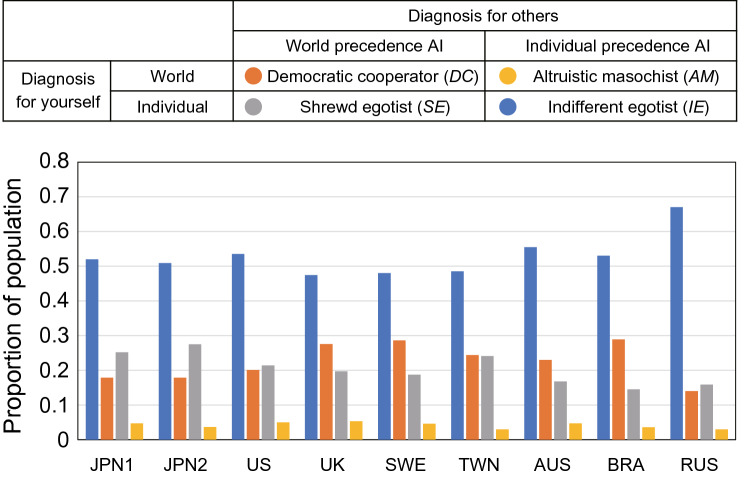
The proportion of the population in each of the four principal categories in each country/area. There are four combinations of answers to which AI you would like to receive a diagnosis from when you or someone else (stranger) becomes sick. The most common answer in all countries/areas was that both they and strangers should be diagnosed by an Individual-AI, *IEs* (blue). In contrast, people who chose for both themselves and strangers to be diagnosed with World-AI, *DCs* (orange). *SEs* (gray) embody the social dilemma in which they want strangers to have a World-AI diagnosis but want an Individual-AI diagnosis for themselves. Those who were tolerant enough to allow strangers to use Individual-AI while they themselves were diagnosed with World-AI, *AMs* (yellow).

The results of the surveys are as follows (Fig. 2, Table S2). *DCs* made up 14.0–28.9% of the respondents. The highest proportion of *DCs* was 28.9% in Brazil, and the lowest was 14.0% in Russia. *SEs* constituted 14.5–27.5% of the sample. The highest proportion of *SEs* was 27.5% in Japan (the second survey), and the lowest was 14.5% in Brazil. *AMs* accounted for 3.0–5.3% of the sample. *IEs* constituted 47.4–67.0% of the sample, the largest proportion among the four categories. The highest proportion of *IEs* was 67.0% in Russia, and the lowest was 47.4% in the United Kingdom.

**Figure 3:**
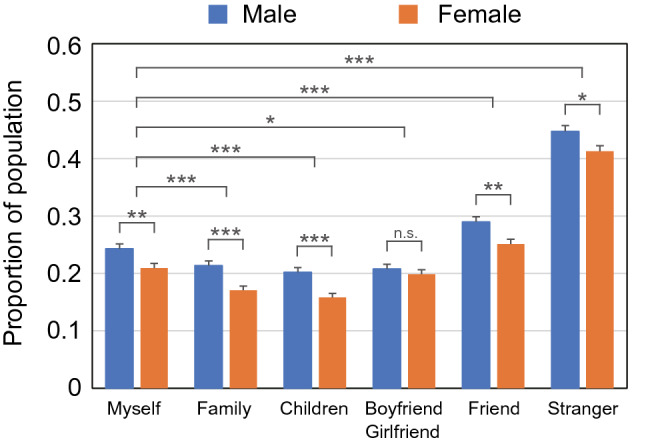
Preference of World-AI for each diagnostic target in the first survey in Japan. Significant gender differences between males (blue) and females (orange) are indicated by asterisks for some diagnostic targets. The respondents’ choice of World-AI diagnosis for themselves and others differed significantly. Note that the respondents chose a World-AI diagnosis for their family, children, and significant others less often than for themselves but chose this diagnosis form more often for their friends and strangers than for themselves. See also Fig. S1 and Tables S3 and S4 for detailed results in other countries/areas. We examined these differences in each response by performing Pearson’s chi-square test with Yates’ continuity correction. Significant codes are shown as follows: ‘*’ as *p* < 0.05, ‘**’ as *p* < 0.01, ‘***’ as *p* < 0.001, and ‘n.s.’ for insignificance.

We analyzed the preference and acceptance of World-AI for each diagnostic target, e.g., myself, family, children, significant other (boyfriend/girlfriend), friend, and stranger. It is noted that systematic differences were found between genders: female preference for Individual-AI was stronger than male preference for Individual-AI in all samples (Figs. 3 and S1). We also found systematic differences in AI preferences for oneself and strangers in all samples (Figs. 3 and S1, and Tables S3 and S4). Among both genders and all countries/areas, the proportion selecting World-AI for their own diagnosis was less than 40%, ranging between 14.6 (in Russian females) and 34.9% (in Swedish males) (Fig. S1, Table S3). However, the proportion selecting World-AI for strangers’ diagnoses was ca. 10–25% higher than that selecting World-AI for their own diagnosis, ranging between 25.9 (in Russian females) and 53.9% (in Taiwan males) (Fig. S1, Table S3). In all counties/areas, the respondents showed a large significant difference in diagnostic AI preferences between strangers and those close to them, including themselves (i.e., family, children, boyfriends/girlfriends), but they exhibited far fewer differences among themselves and those closest to them. Interestingly, in five countries (Japan, the United Kingdom, Sweden, Brazil, and Russia), they choose Individual-AI for their children’s diagnoses more often than for their own (Figs. 3 and S1, and Table S4). The AI preferences for friends were located between those of strangers and themselves and were usually closer to the latter (Figs. 3 and S1). For both genders, the AI preferences for the respondents and their significant others were not different, except in Sweden and Japan, where the respondents were more likely to choose Individual-AI for the significant others (Fig. S1, Table S4).

**Figure 4:**
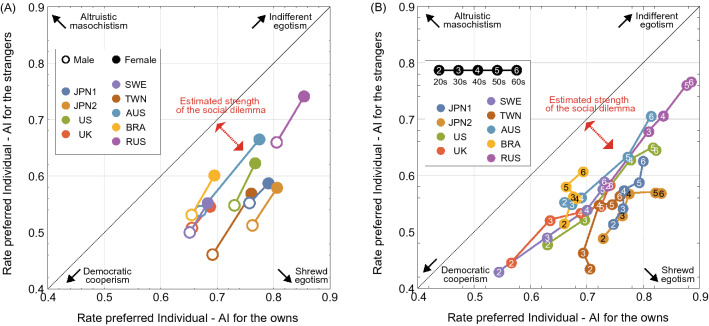
Country/area, gender and age differences in the social dilemma and characteristics of the responses. Scatterplot of the proportion of the sample who chose the Individual-AI diagnosis over the World-AI diagnosis. The horizontal axis is the portion of the sample who preferred Individual-AI to diagnosis themselves, and the vertical axis is that for strangers. The upper-right region is an individualistic response in which the respondents preferred the Individual-AI diagnosis for both themselves and strangers. In contrast, the lower-left region is an institutionalized response in which the respondents preferred the World-AI diagnosis when either themselves or strangers get sick. The lower-right region below the diagonal indicates that the respondents’ preference for the Individual-AI diagnosis was higher for themselves than for strangers. The distance between the diagonal and the plot shows the estimated strength of the social dilemma. (**A**) Gender differences in all eight countries/areas and (**B**) age group differences in all eight countries/areas. The absence of plots in the upper left region means that the respondents judged their own lives to be more important than those of strangers in general.

A scatter plot representing the potential risk of AMR and the strength of the social dilemma estimated in each country/area is shown in Fig. 4. Here, the lower-right region below the diagonal indicates that the respondents’ preference for the Individual-AI diagnosis was higher for themselves than for strangers. We can consider the distance between the diagonal and the plot as the ‘estimated’ strength of the social dilemma. Note that, this is not mathematical definition of a social dilemma strength^17^. However, in the dilemma situation such as the current topic, where the payoff matrix in the real world is unknown, this would be one of the most efficient methods to estimate social dilemma strength. As a result, since all response plots are located in the region below the diagonal, the social dilemma in the use of antimicrobials and AMR risks are at least recognized by some people, both male and female and younger and older in all countries/areas (Fig. 4). Interestingly, more male respondents than female respondents were *DCs* (lower-left plot in figure) (Fig. 4A). In other words, females are more likely than males to be *IEs* (upper-right plot in figure) and are more tolerant of themselves and strangers. The younger age groups were more likely to be *DCs* than the older age groups in all countries/areas (Fig. 4B). Our analysis shows that the proportion of respondents who chose Individual-AI for their own and strangers’ diagnoses significantly varied with gender and age in all countries/areas (Figs. S2 and S3). In other words, the females and older respondents in the same country/area were more likely to choose Individual-AI in diagnosing themselves and not strangers than were the male and younger respondents.

## Discussion

Our results reveal that the promoters of the World-AI (*DCs*) and those who recognize the social dilemma (*SEs*) account for approximately 15–30% of the samples, depending on countries/areas, while approximately 50–70% of the samples exhibited the ignorance of AMR (*IEs*); few were *AMs* (Fig. 2, Table S2). A large proportion of the respondents (almost half to 70%) did not prefer the World-AI, which may implicate a bleak future for AMR control. Nevertheless, we have two positive findings regarding the World-AI. One is that 15–30% of the respondents (*DCs*) chose the World-AI. They were willing to tackle the issue of AMR in a positive way, at least on the surface. The second is that another 15–30% recognized the social dilemma (*SEs*). They recognize that the excess use of antimicrobials is a social dilemma (problem) (for a more detailed analysis, see Supplementary Information).

We conducted two Japanese surveys before and during the COVID-19 crisis (JPN1: Jan. 8–10, 2020 and JPN2: Jul. 1–7, 2020), expecting differences in public recognition of social dilemmas in public health. These surveys in Japan showed no temporal differences due to the effects of COVID-19 (Table S5). These two surveys retrospectively indicate the stability of public opinion in the current questionnaires. This also indicates the difficulty of spreading the recognition of the AMR problem, and every educational effort is needed to make people understand this serious public health problem.

In the current survey, we introduce two different AIs to compare the preferences to keep the comparisons between countries/areas^15,16^. However, the introduction of AIs might have introduced the deviations because of the differences in the perceptions of AI among countries/areas. Some ideal alternative framing should be considered in the questionnaire in future studies, e.g., physicians instead of AIs.

Some studies have noted the existence of a social dilemma behind the spread of AMR^7–12^. It is noteworthy that previous studies have focused on the social dilemma at the level of policy makers and medical workers but not at the level of the general public or citizens. For example, some games developed in previous studies are not a game involving patients but games involving antimicrobial prescription by gatekeepers (physicians)^10,12^. Here, we specifically turn our attention to the social dilemma that exists within the general public, which accounts for the majority of patients. For this reason, we successfully observed the recognition of social dilemmas by presenting the problem of diagnostic AI dissemination for use when the respondent or others become sick.

We should also note that the current topic is slightly different from the social dilemma regarding the excess use of antimicrobials for animal breeding (livestock production). Some antimicrobials are used in excess in livestock (cattle, pigs, chicken, fish, etc.) to marginally accelerate their growth and avoid accidental infections^18^. Animal breeders who use antimicrobials (i.e., free riders) gain more benefit than those who refrain from using antimicrobials. This can be considered a typical tragedy of the commons^19^. Soon after the recognition of this problem, the European Union (EU) introduced a ban on growth-promotion antimicrobials in livestock more than a decade ago^19^. As an economic problem, tragedies of the commons occur in various fields of food production and renewable resources. On the other hand, issues related to human health are not purely economic activities but are ethically more complex. Therefore, government should intervene only with extreme caution^7^. Thus, although a dilemma with a similar tragedy of the commons structure, we expect that the AMR problem cannot be solved as easily as the problem of livestock production, although the latter is still a difficult problem. This is the difference between the current topic and the animal agriculture dilemma.

The social dilemma in control measures of infectious disease, such as nonpharmacological intervention and vaccination, is already well known and well recognized in public. For example, COVID-19 vaccines have become a central issue worldwide. However, in many countries, it takes longer to reach the level of herd immunity because considerable proportions of people are suspicious about the vaccination or worried about serious adverse effects^20,21^. As shown in Fig. 1, infectious disease control will collapse as the number of free riders increases and the number of cooperators decreases in a chain reaction. Therefore, preventing the emergence of free riders by resolving social dilemmas is an extremely important theme in public health. In fact, the importance of the social dilemma related to vaccination has already been recognized, and vaccination games in game theory have been extensively studied^22,23^. In the vaccination game, the ‘herd immunity’ created by voluntary vaccination behavior is common. On the other hand, the social dilemma concept we present in the current paper considers ‘repertoire of effective antimicrobials’ and the ‘public health’ itself as commons. In addition, the game structure presented in this paper has a different timescale from that of the vaccination game, which usually assumes seasonal disease outbreak because it is a game that has been going on since the spread of antimicrobials. Thus, the social dilemma in excess use of antimicrobials is another case of a game situation in the public health field.

Our results also indicate that the use of antimicrobials is important for people beyond its mere economic impact. If people were rational for their selfish benefit in the short term, all people would be *SEs* (i.e., *SEs* would make up 100% of the respondents). However, the results indicate that most of the people did not choose the most rational option in the short term (*SEs* represented only 15–30% of the respondents). Thus, our survey reveals an important perspective. Most people are aware that their own health is a completely private issue, even though their overall health is a public issue. Furthermore, most people (*IEs*) recognize and respect the health of a stranger as purely that individual’s private good. However, we know that free economic behavior (profit maximization) on private (not public) lands also leads to resource depletion^24,25^. Therefore, the development of incentives for refraining from the overuse of antimicrobials is an important research topic. It is worth to consider the combinations of the various incentive implementation methods, e.g., rewards and penalties^26^, early exclusion of free rider^27^, optimization of incentives^28^, fixed and flexible incentives^29^. Here, key elements in resolving this issue are sharing ‘correct knowledge’ and ‘a sense of crisis,’ and setting some specific goal^30^. Our findings indicate that one of the essential problems in infectious disease is rooted in the fact that the health of individuals belongs to private and public simultaneously.

## Methods

### Design concept of the survey

We designed the questionnaire so that we could observe this social dilemma using two types of imaginary artificial-intelligence (AI) physicians who operate from either an individual or societal perspective. This setting allows us to the ask the respondents whether they recognize the social dilemma without using a direct question, including the explanation of this dilemma. We then ask which AI physicians they would choose for themselves as patients. This method also eliminates the patient’s dependency on physicians’ prescription decisions.

We assume two AI medical diagnosis systems: “Individual precedence AI” (abbreviated Individual-AI) and “World precedence AI” (abbreviated World-AI). Both AIs diagnose and prescribe medicine automatically. The Individual-AI system diagnoses patients and prescribes medicine to prevent infections based on an individual perspective, including all prophylactic prescriptions against rare accidental infections (not yet present and unlikely to occur). It does not consider the global risk of AMR in the decision. The World-AI system, instead, takes into account the global mortality rate of AMR, aiming to reduce the total number of all AMR-related deaths. Because of this, this AI system does not prescribe antimicrobials against rare and not-yet-present infections. These assumptions are not explicitly described in the questionnaire and the two types of AIs are simply presented: one does not consider AMR, and the other does because we intend to collect personal naïve intuitive choices to avoid instant logical derivations while answering the questionnaire.

We ask the respondents which AI system they favored for themselves, their family, children, significant others, common friends, and strangers. This questionnaire design allows us to observe the social dilemma. For example, it shows a typical social dilemma caused by preferring the use of Individual-AI for diagnosing oneself but preferring the use of World-AI for diagnosing strangers. We may be able to estimate how many people consciously or unconsciously recognize (express) this social dilemma and the corresponding potential risk of AMR emergence/spread in a society. If many respondents choose Individual-AI for diagnosis over World-AI, the risk of AMR emergence/spread is expected to be higher. Note that the design of the questionnaire was inspired by a similar web survey study that focused on the social dilemma in autonomous vehicles (AVs) under the situation of the trolley problem^15,16^. They asked respondents which AV they would like to be more widespread, assuming two AVs: one that protects pedestrians and sacrifices passengers and the other that protects passengers and runs over pedestrians. They demonstrated that there is a social dilemma in the widespread use of AVs, as the respondents indicated that they want their AVs to protect passengers first but that they want AVs used by others to protect pedestrians first.

### Recruitment

The survey entitled “Survey on Medical Advancement” was administered to 8 countries/areas. The survey was conducted 4 times (Table S1).

For the two surveys in Japan, an internet survey company, Cross Marketing Inc. (https://www.cross-m.co.jp/en/), created the questionnaire webpages based on our study design. The company also collected the data. As of April 2020, Cross Marketing Inc. has 4.79 million people in an active panel (survey participants who registered in advance). Here, the definition of an active panel is a survey respondent who has been active within the last year. For the panels, the questionnaire and response column were displayed on the website through which the respondents could complete and submit their responses. We extracted 500 submissions for each gender and each age group by random sampling from all samples collected during the survey periods. The surveys in the 7 countries/areas (i.e., the United States, the United Kingdom, Sweden, Taiwan, Australia, Brazil, and Russia) are conducted by Cint (https://www.cint.com/). Cint is the world’s largest consumer network for digital survey-based research. The headquarters of the company is in Sweden. Cint maintains a survey platform that contained more than 100 million consumer monitors in over 80 countries as of May 2020. For surveys in the US, UK, Sweden, Taiwan, Australia, Brazil, and Russia, Cint Japan (https://jp.cint.com/), which is the Japanese distributor of Cint, created translated questionnaire webpages based on our study design. The company also collected the data. We extracted at least 500 (US, UK, SWE, BRA, RUS) or 250 (TWN, AUS) submissions for each gender (male and female) and each age group (20 s, 30 s, 40 s, 50 s, and 60 s) by random sampling from all samples collected between survey periods.

Note that both companies eliminated inconsistent or apathetic respondents. For example, respondents with inconsistent responses (e.g., the registered age of the respondent differed from the reported age at the time of the survey.) were eliminated before reaching the authors. In addition, respondents with significantly short response times (i.e., shorter than 1 min) were eliminated because they may not have read the questions carefully.

### Questionnaire

At least 5000 (2500 male and 2500 female) responses in total were received from individuals in their 20 to 60 s for each of Japan, the U.S., the U.K., Sweden, Brazil, and Russia. There were at least 500 male and 500 female respondents in each age group (i.e., 500 male and 500 female participants in their 20 s, 500 male and 500 female participants in their 30 s…, 500 male and 500 female participants in their 60 s). In Taiwan and Australia, half of the responses (i.e., 2,500) were collected in each age group because these countries/areas have small populations. The respondents provided their anonymous personal information (gender and age) and answered the following question:

Choose one item regarding AI diagnosis in the following.

When (1) you, (2) your family (parents, brother, sister, husband, or wife), (3) your children, (4) your boy/girlfriend, (5) your friend, and (6) a stranger get sick, which would you like (him/her) to use the World precedence AI or the Individual precedence AI?

‘World precedence AI’ or ‘Individual precedence AI’

All respondents provided informed consent before completing the questionnaire. The respondents were rewarded with electronic points that could be exchanged for cash, gift certificates, frequent flyer miles or electronic money (e-money) for various services. The exact amount of the electronic point reward is unknown due to the company’s privacy policy. This study was approved by the Ethical Committee of the Institute of Tropical Medicine, Nagasaki University (Approval No. 190619217). All methods were performed in accordance with the relevant guidelines and regulations. The questionnaire web pages used in the current survey can be found in the Supplementary Information.

### Statical analysis

We examined the respondents’ differences in AI preference for themselves and others by performing Pearson’s chi-square test with Yates’ continuity correction (Figs. 3, S1 and Tables S4, S5). Significance codes are as follows: ‘*’ as *p* < 0.05, ‘**’ as *p* < 0.01, ‘***’ as *p* < 0.001, and ‘n.s.’ for insignificance. We also examined gender and age group differences by logistic regression analysis (Figs. S2, S3). The software used for each analysis was R (ver. 4.0.2) and R studio.

## Supplementary Information

Supplementary Information.

## Data Availability

All data are available in the main text or the Supplementary Information.
