# Supplementary material for: Social dilemma in the excess use of antimicrobials incurring antimicrobial resistance
Source: Sci Rep. 2022 Dec 6;12:21084. doi: 10.1038/s41598-022-25632-1 (PMC9726979; doi:10.1038/s41598-022-25632-1)
Supplement: Supplementary file 1 — Supplementary Information. [file 41598_2022_25632_MOESM1_ESM.pdf]

Supplementary Information for
Social dilemma in the excess use of antimicrobials incurring antimicrobial resistance

Hiromu Ito, Takayuki Wada, Genki Ichinose, Jun Tanimoto, Jin Yoshimura, Taro Yamamoto, Satoru Morita

Methods

Questionnaire web page
Cint Japan created the translated questionnaire webpages based on our study design. The following URL shows the actual web page used for the survey. A total of 4 questions are displayed in our survey. However, in this manuscript, we focus on only the first question:

Choose one item regarding AI diagnosis in the following.

When (1) you, (2) your family (parents, brother, sister, husband, or wife), (3) your children, (4) your boy/girlfriend, (5) your friend, and (6) a stranger get sick, which would you like (him/her) to use the World precedence AI or the Individual precedence AI?

'World precedence AI' or 'Individual precedence AI'

The entire questionnaire is displayed below, including the above question.

▼USA
Overview
https://d8aspring.post-survey.com/preview/previewPageAll.php?key=Z2zvoUTO
Demo
https://d8aspring.post-survey.com/preview/index.php?key=Z2zvoUTO&lang_c=ja

▼UK
Overview
https://d8aspring.post-survey.com/preview/previewPageAll.php?key=IQhRORK5
Demo
https://d8aspring.post-survey.com/preview/index.php?key=IQhRORK5&lang_c=ja

▼Sweden
Overview:
https://d8aspring.post-survey.com/preview/previewPageAll.php?key=7EagCMPq
Demo:
https://d8aspring.post-survey.com/preview/index.php?key=7EagCMPq&lang_c=ja

▼Taiwan
Overview:
https://d8aspring.post-survey.com/preview/previewPageAll.php?key=1Sl3dSUJ
Demo:
https://d8aspring.post-survey.com/preview/index.php?key=1Sl3dSUJ&lang_c=ja

▼Australia
Overview:
https://d8aspring.post-survey.com/preview/previewPageAll.php?key=hv6drWm6
Demo:
https://d8aspring.post-survey.com/preview/index.php?key=hv6drWm6&lang_c=ja

▼Brazil
Overview:
https://d8aspring.post-survey.com/preview/previewPageAll.php?key=Ln1Iumxc
Demo:
https://d8aspring.post-survey.com/preview/index.php?key=Ln1Iumxc&lang_c=ja

▼Russia
Overview:
https://d8aspring.post-survey.com/preview/previewPageAll.php?key=s8jOCQ5T
Demo:
https://d8aspring.post-survey.com/preview/index.php?key=s8jOCQ5T&lang_c=ja

Questionnaire layouts (e.g., USA ver.)

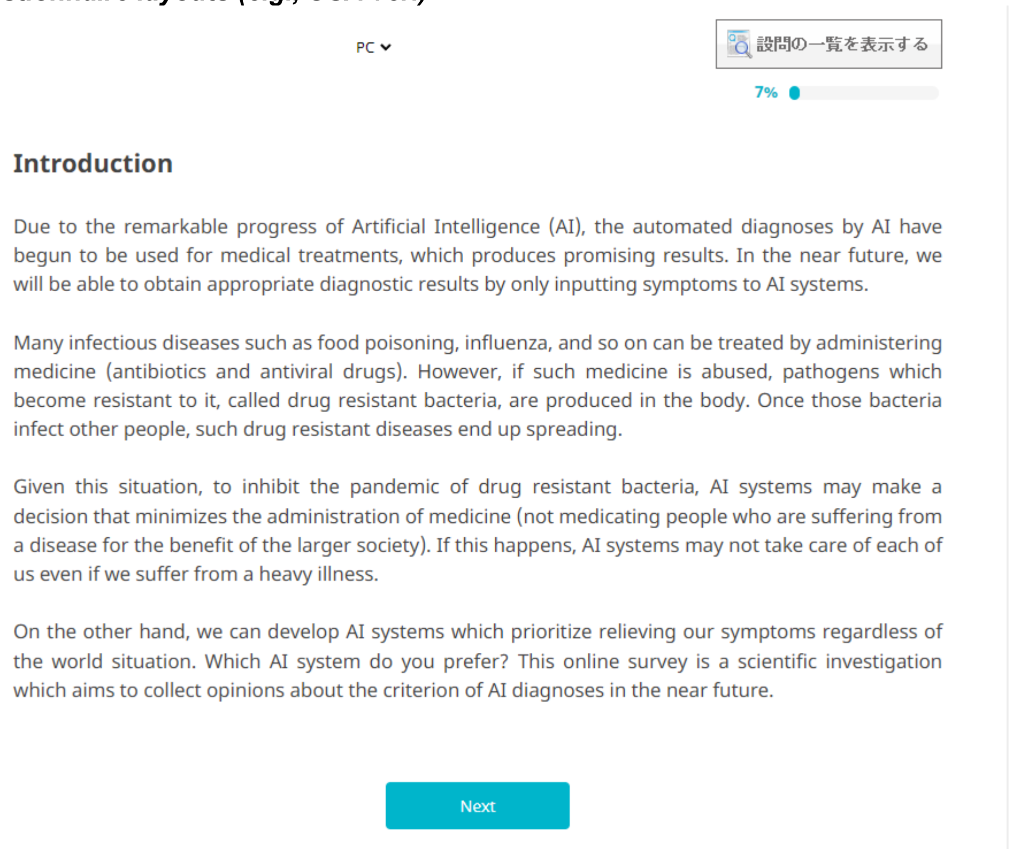
PC

設問の一覧を表示する

7%

Introduction

Due to the remarkable progress of Artificial Intelligence (AI), the automated diagnoses by AI have begun to be used for medical treatments, which produces promising results. In the near future, we will be able to obtain appropriate diagnostic results by only inputting symptoms to AI systems.

Many infectious diseases such as food poisoning, influenza, and so on can be treated by administering medicine (antibiotics and antiviral drugs). However, if such medicine is abused, pathogens which become resistant to it, called drug resistant bacteria, are produced in the body. Once those bacteria infect other people, such drug resistant diseases end up spreading.

Given this situation, to inhibit the pandemic of drug resistant bacteria, AI systems may make a decision that minimizes the administration of medicine (not medicating people who are suffering from a disease for the benefit of the larger society). If this happens, AI systems may not take care of each of us even if we suffer from a heavy illness.

On the other hand, we can develop AI systems which prioritize relieving our symptoms regardless of the world situation. Which AI system do you prefer? This online survey is a scientific investigation which aims to collect opinions about the criterion of AI diagnoses in the near future.

Next

Page 1: Introduction

PC

設問の一覧を表示する

13%

Confirmation of consent

We would like to know what you think about the criterion of AI diagnoses. Answering the survey takes about 10 minutes. Cooperating with our survey is up to you. When you are answering questions, you can stop answering at any time if you would like to withdraw. Note that you will forfeit your survey incentives for this project in that case.

We do not use the information obtained from the survey other than in this investigation. The web research company destroys the retained data after it delivers the data to us so that personally identifiable information and survey information cannot be recovered. The answer data is stored and analyzed after randomizing the respondent ID not to connect to the respondent. Thus, it is impossible to connect the survey information to personally identifiable information even in the case of an information leak.

This study was approved by the local ethical committee in the Institute of Tropical Medicine Nagasaki University, Japan on July 30, 2019.

I voluntarily agree to participate in this study.

- Yes
- No

Next

Page 2: Confirmation of consent

PC

設問の一覧を表示する

20%

Scenario

Assume that the AI system has the following two diagnosis criteria:

1) World precedence AI

This AI mainly considers the world situation, but also your symptoms. For instance, drug resistant bacteria prevailing in the world spread by drug abuse. Considering this situation, the AI system may make a decision that it is better for you to sleep at home than prescribing medicine even if you complain of pain. In fact, you would recover from an illness by sleeping at home, but the recovery time may be longer than if you are prescribed medicine. It also raises the risk that your family will be infected.

2) Individual precedence AI

This AI only considers your symptoms and makes the most effective decision for alleviation and treatment of the symptoms. For instance, this AI does not consider the present situation where drug resistant bacteria have spread all over the world. If this AI diagnoses that it is better to prescribe medicine than to sleep at home in order to recover from illness as soon as possible, this AI does so.

Next

Page 3: Explanation of scenario

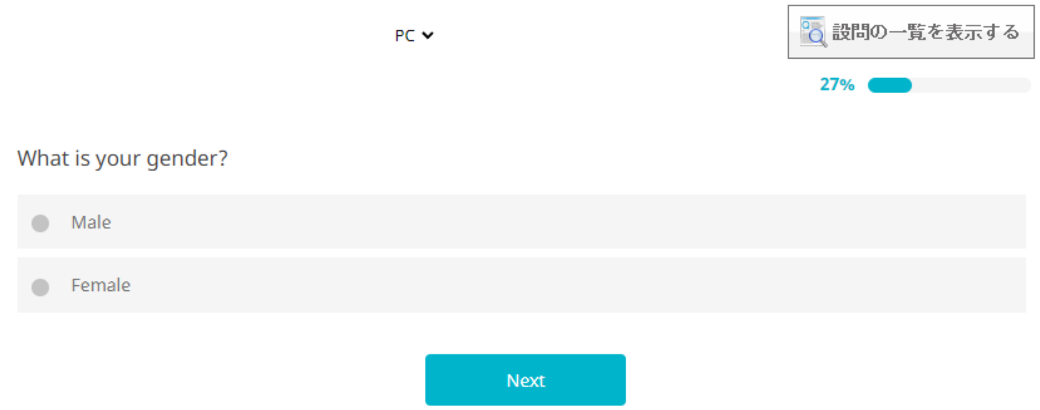

Page 4: Personal information input (Gender)

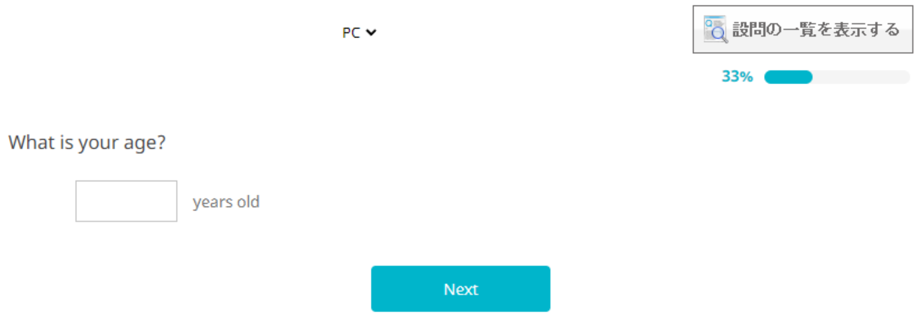

Page 5: Personal information input (Age)

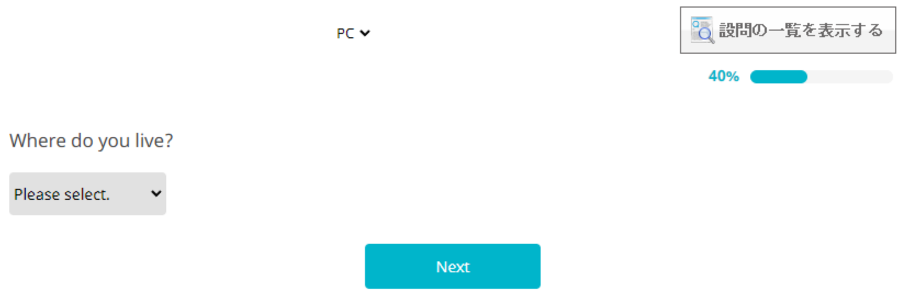

Page 6: Personal information input (State/province level address)

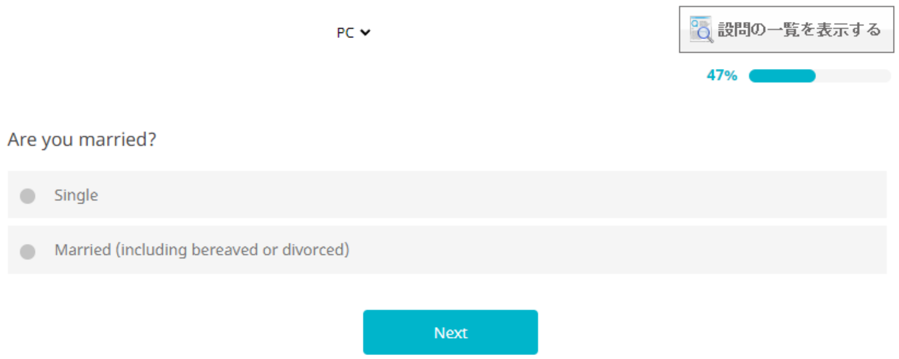

Page 7: Personal information input (Marital status)

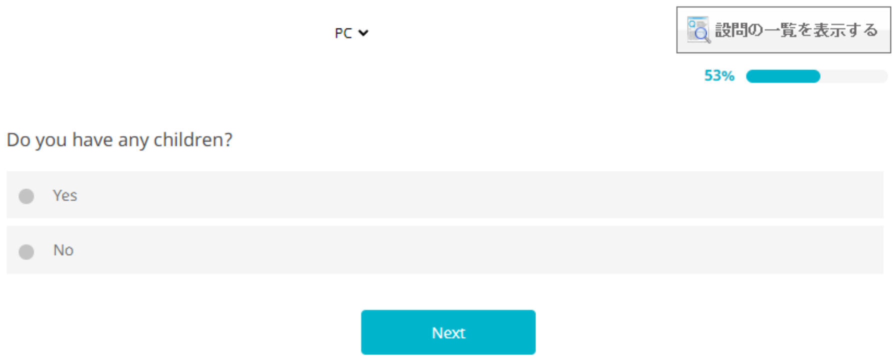

Page 8: Personal information input (Existence of children)

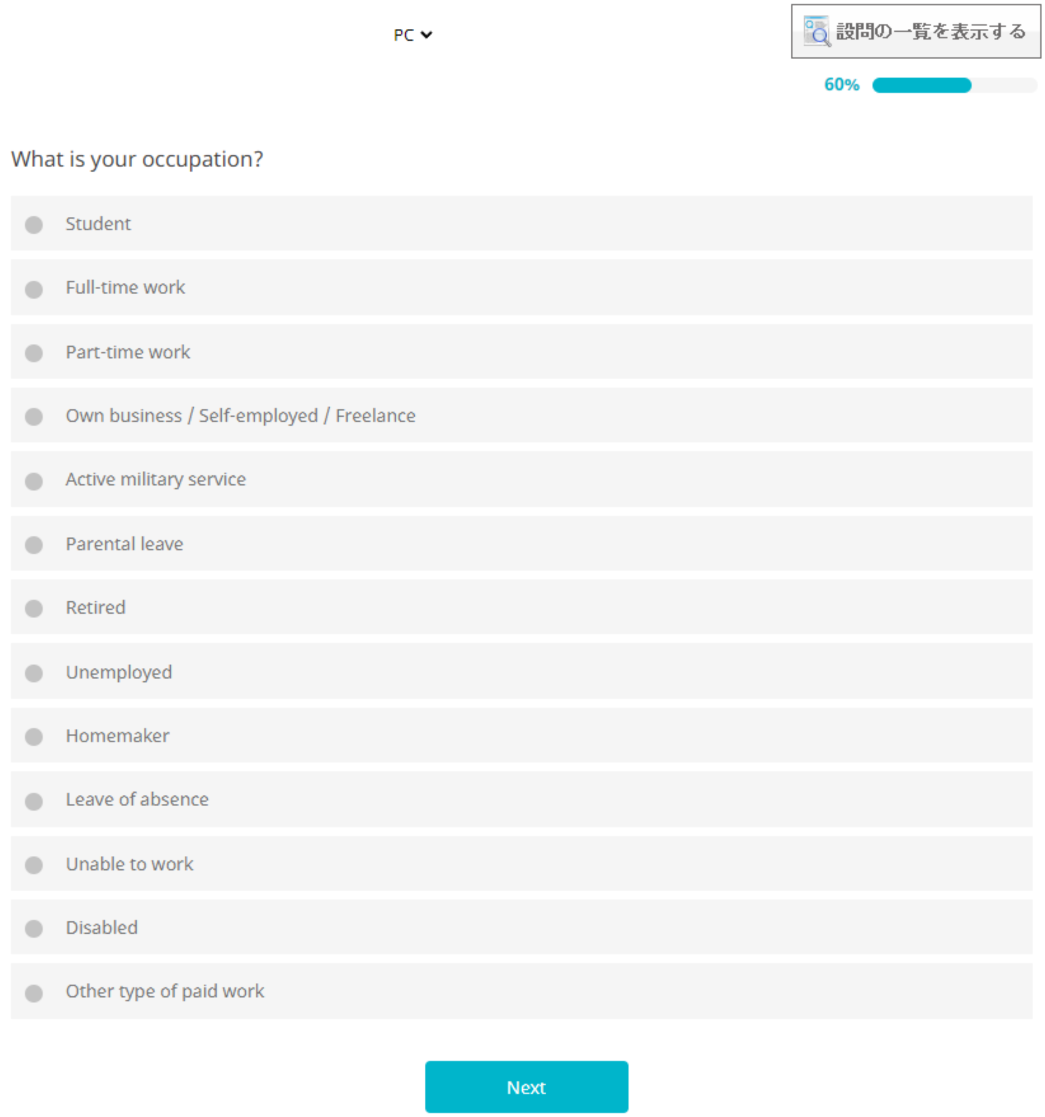

Page 9: Personal information input (Occupation)

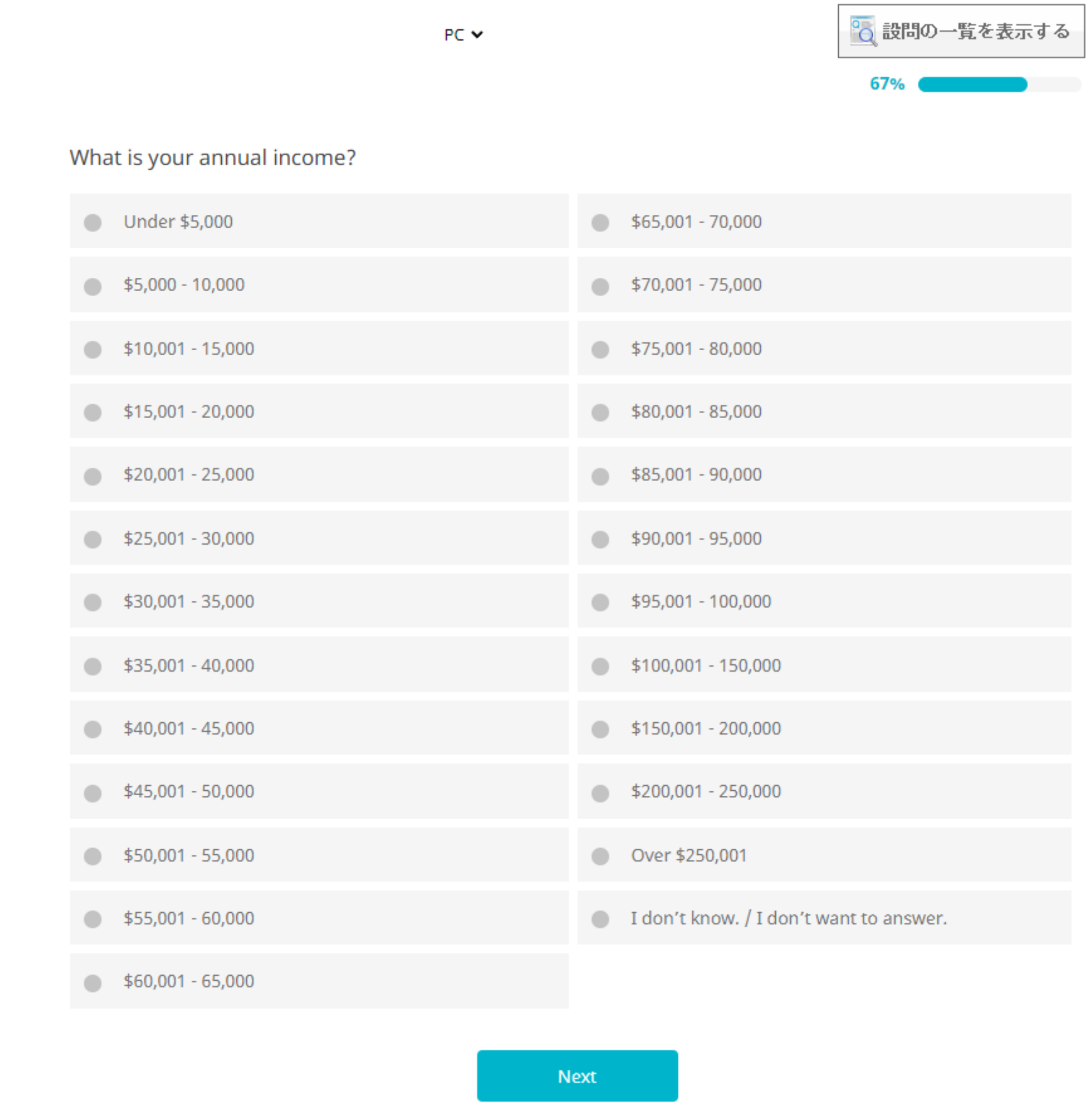
PC
設問の一覧を表示する
67%

What is your annual income?

Under $5,000	$65,001 - 70,000
$5,000 - 10,000	$70,001 - 75,000
$10,001 - 15,000	$75,001 - 80,000
$15,001 - 20,000	$80,001 - 85,000
$20,001 - 25,000	$85,001 - 90,000
$25,001 - 30,000	$90,001 - 95,000
$30,001 - 35,000	$95,001 - 100,000
$35,001 - 40,000	$100,001 - 150,000
$40,001 - 45,000	$150,001 - 200,000
$45,001 - 50,000	$200,001 - 250,000
$50,001 - 55,000	Over $250,001
$55,001 - 60,000	I don't know. / I don't want to answer.
$60,001 - 65,000	

Next

Page 10: Personal information input (Income)

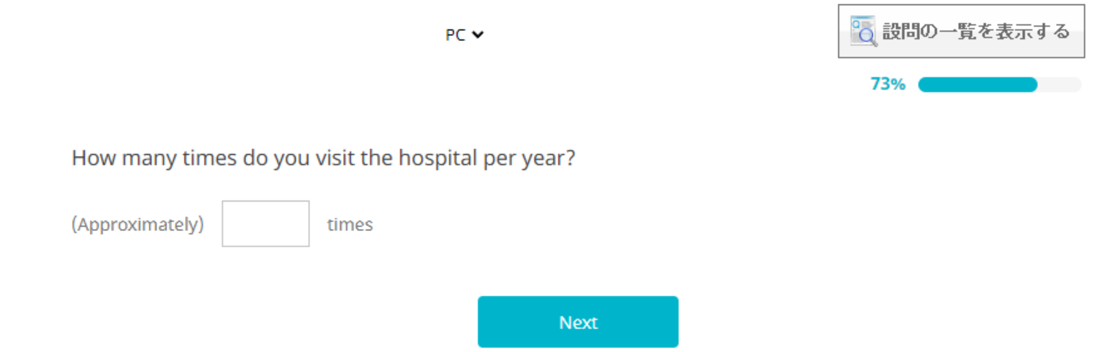
PC
設問の一覧を表示する
73%

How many times do you visit the hospital per year?

(Approximately) times

Next

Page 11: Personal information input (Frequency of hospital use)

PC

設問の一覧を表示する

80%

Choose one item regarding AI diagnosis in the following.

1.) When you get sick, which would you like to use the World precedence AI or the Individual precedence AI?

- World precedence AI
- Individual precedence AI

2.) When your family (parents, brother, sister, husband, or wife) gets sick, which would you like them to use the World precedence AI or the Individual precedence AI?

- World precedence AI
- Individual precedence AI

3.) When your children get sick, which would you like to use the World precedence AI or the Individual precedence AI?

- World precedence AI
- Individual precedence AI

Page 12: AI preference for each diagnostic target

PC

設問の一覧を表示する

87%

How common, based on percentage, would you like the World precedence AI and the Individual precedence AI, respectively, to become? The total penetration ratio must be 100%.

World precedence AI: ____ %

Individual precedence AI: ____ %

Total : 0

Next

Page 13: AI preference when two types of AI system are widely used

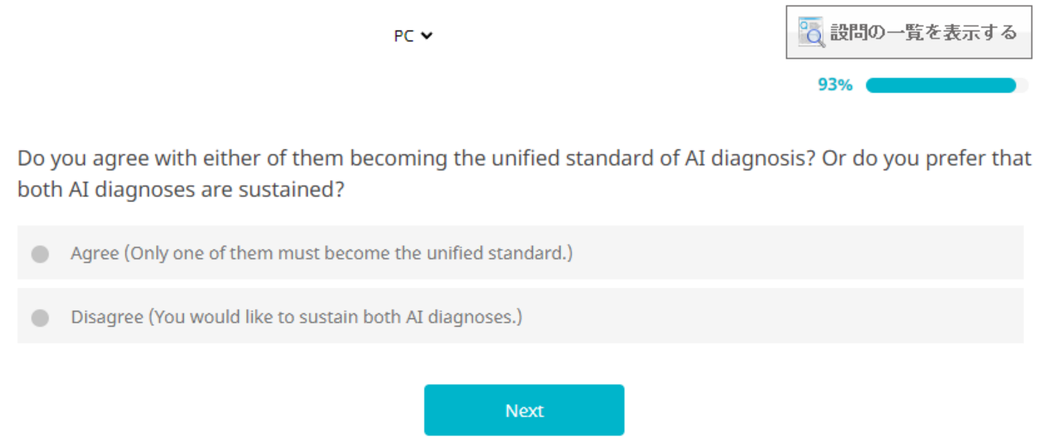

Page 14: Attitude toward AI standardization

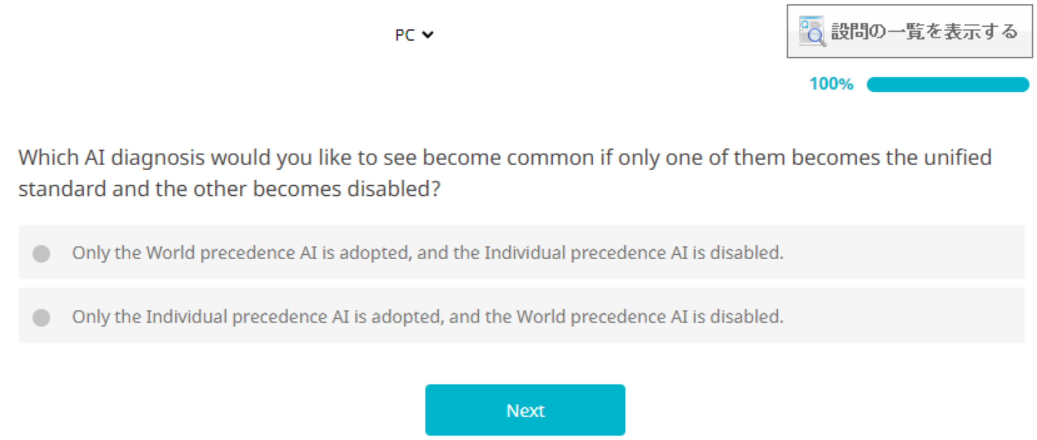

Page 15: Select of AI to be standardized

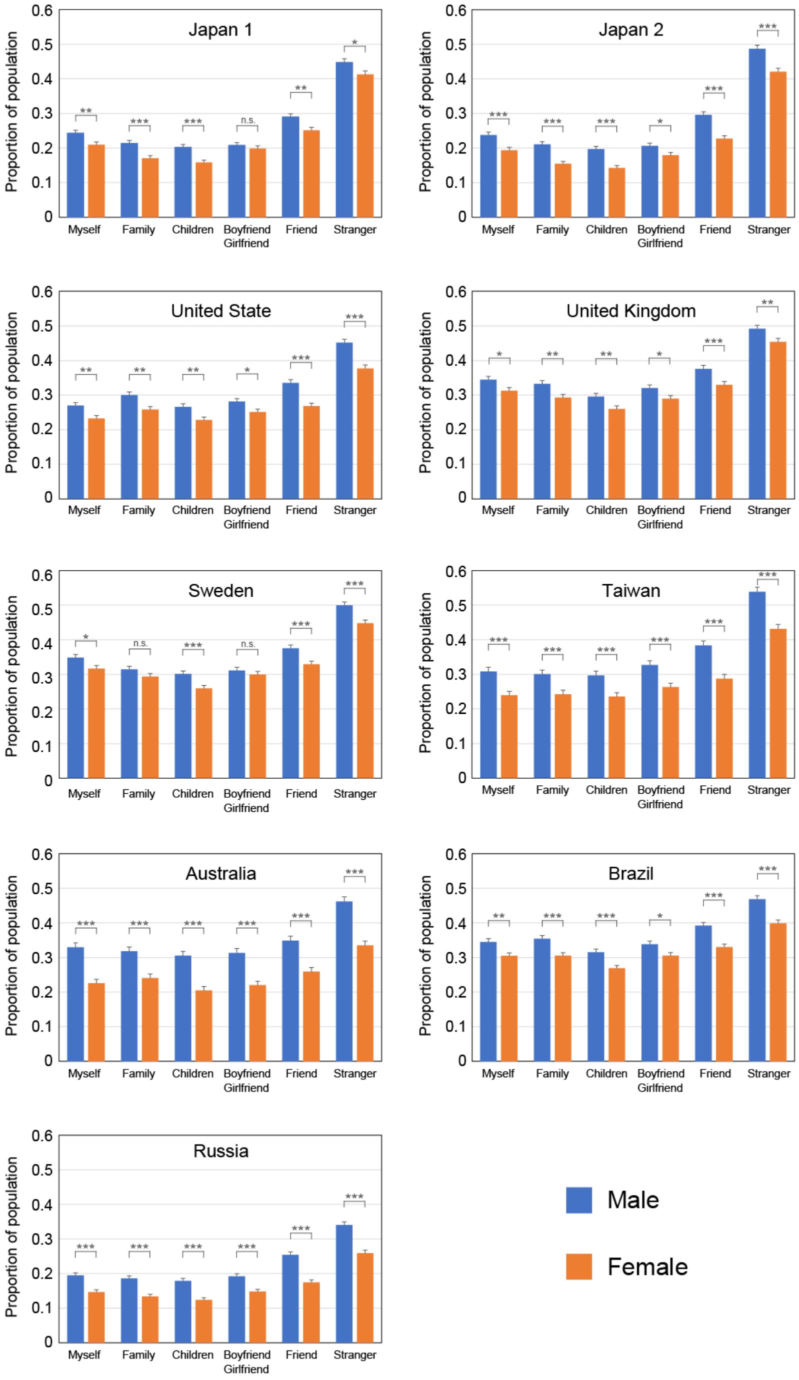

Fig. S1. Preference of World-AI for each diagnostic target. Significant gender differences between men (blue) and women (orange) are shown in asterisks for some diagnostic targets. We examined these gender differences in each response by performing Pearson's chi-square test with Yates' continuity correction. Significance codes are as follows: '*' as $p < 0.05$, '**' as $p < 0.01$, '***' as $p < 0.001$, and 'n.s.' for insignificance.

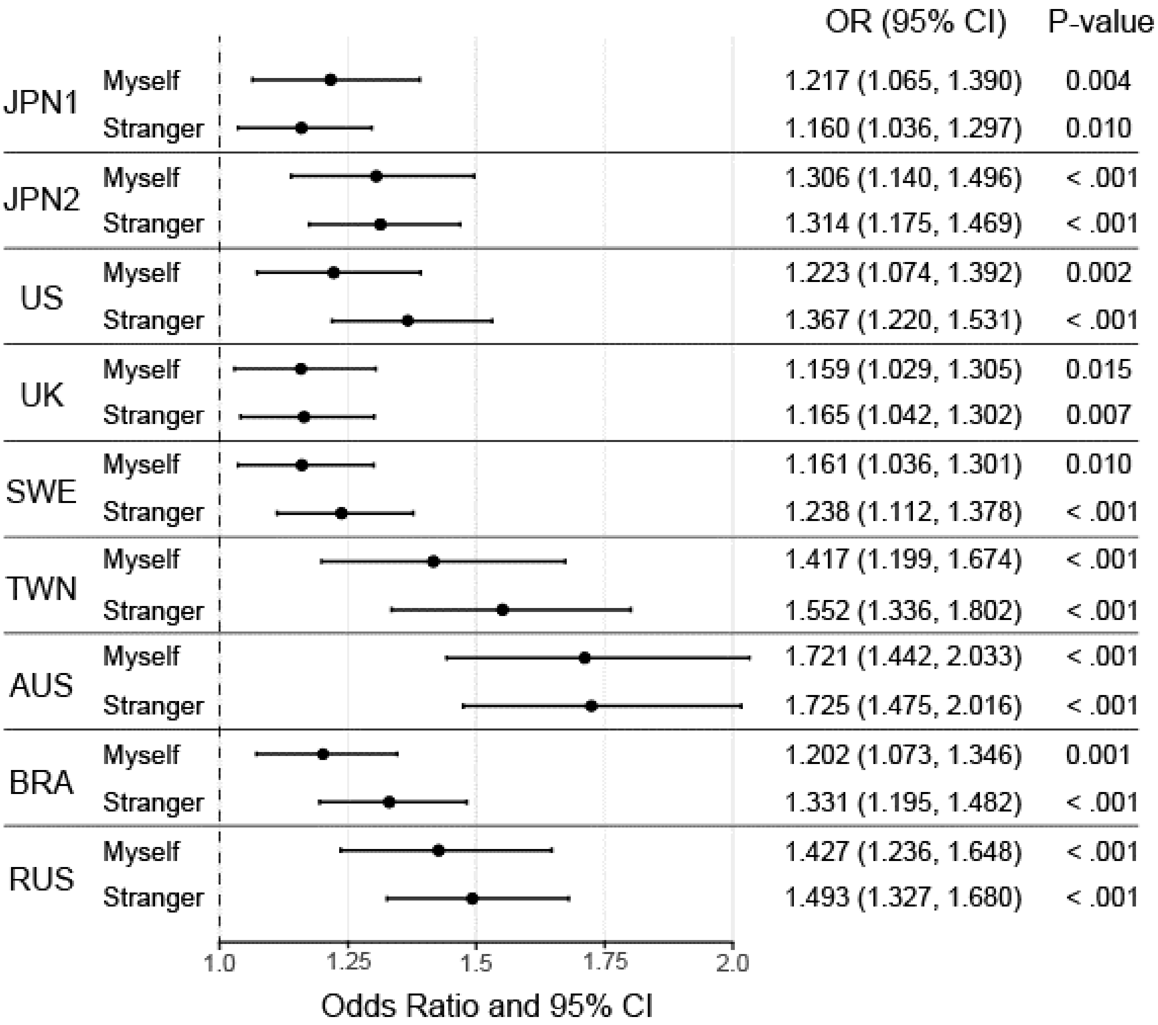

Fig. S2. Odds ratio for logistic regression analysis for Individual-AI diagnosis preference for oneself and strangers by gender. The odds ratios and 95% CIs exceed 1 in all countries/areas. Independent of country/area and diagnostic target (myself and stranger), the female respondents significantly preferred the Individual-AI over male respondents.

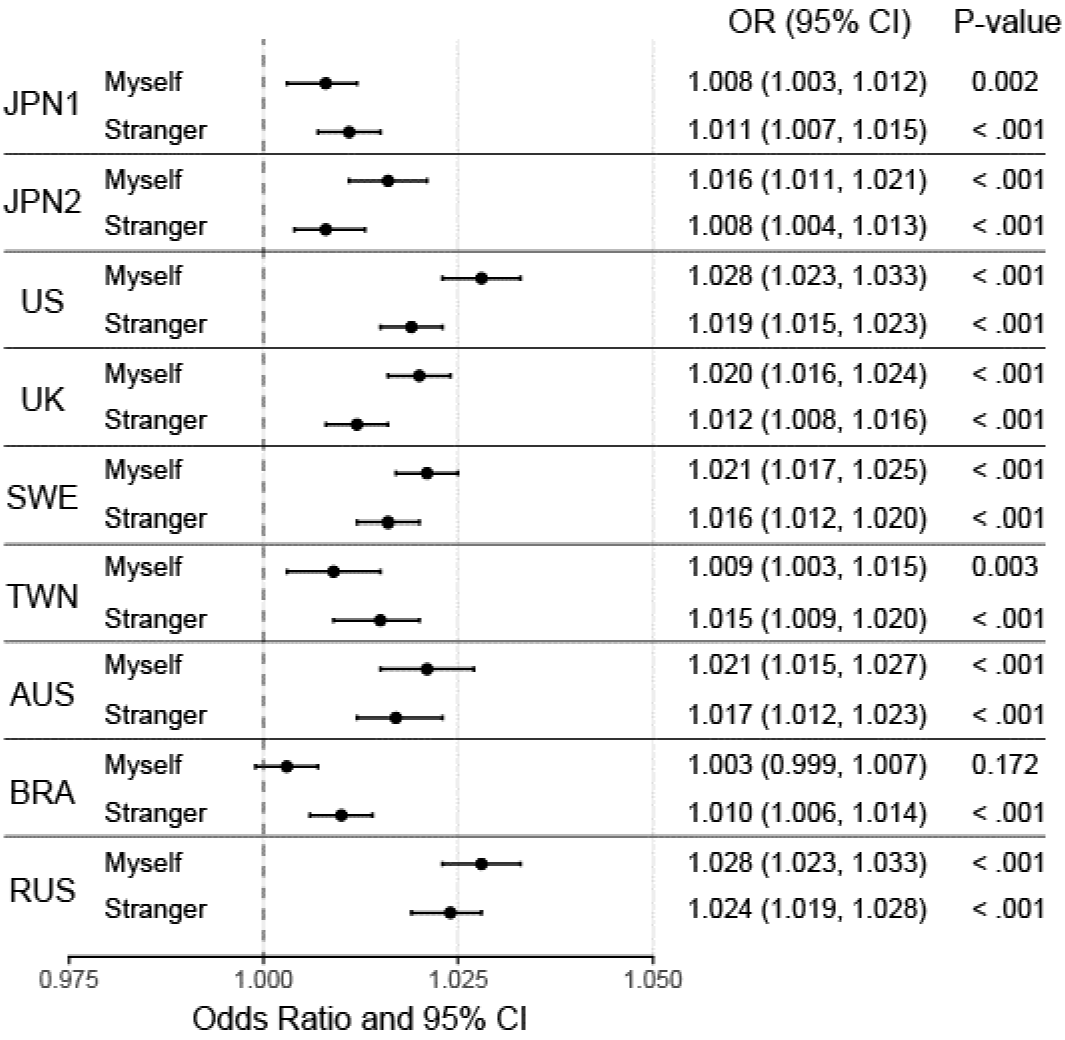

Fig. S3. Odds ratio for logistic regression analysis for Individual-AI diagnosis preference for oneself and strangers by age. In all countries/areas, the odds ratios and 95% CIs exceed 1, except for the diagnosis of oneself in Brazil. Independent of country/area and diagnostic target (myself and stranger), the older respondents significantly preferred Individual-AI over the younger respondents, except for the diagnosis of oneself in Brazil.

Table S1. Basic information of the surveys.

Country/Area	Code	Date of Survey	Total sample size (Male: Female)	Survey Company
Japan 1	JPN1	2020/Jan/8~10	5000 (2500: 2500)	Cross Marketing Inc.
Japan 2	JPN2	2020/Jul/1~7	5000 (2500: 2500)	Cross Marketing Inc.
United States	US	2020/Jul/1~7	5037 (2519: 2518)	Cint Japan
United Kingdom	UK	2020/Jul/1~7	5038 (2517: 2521)	Cint Japan
Sweden	SWE	2021/May/18~26	5446 (2708: 2738)	Cint Japan
Taiwan	TWN	2021/May/18~26	2820 (1405: 1415)	Cint Japan
Australia	AUS	2021/May/18~26	2723 (1353: 1370)	Cint Japan
Brazil	BRA	2021/Jun/23~30	5471 (2726: 2745)	Cint Japan
Russia	RUS	2021/Jun/23~30	5443 (2722: 2721)	Cint Japan

Table S2. The number of respondents in each of the four dilemma categories (%).

Country/Area	Indifferent Egoist	Democratic Cooperator	Shrewd Egoist	Altruistic Masochist
JPN1	2612 (52.2%)	893 (17.9%)	1258 (25.2%)	237 (4.7%)
JPN2	2544 (50.9%)	893 (17.9%)	1377 (27.5%)	186 (3.7%)
US	2695 (53.5%)	1011 (20.1%)	1077 (21.4%)	254 (5.0%)
UK	2390 (47.4%)	1390 (27.6%)	993 (19.7%)	265 (5.3%)
SWE	2615 (48.0%)	1559 (28.6%)	1020 (18.7%)	252 (4.6%)
TWN	1368 (48.5%)	687 (24.4%)	680 (24.1%)	85 (3.0%)
AUS	1510 (55.5%)	626 (23.0%)	458 (16.8%)	129 (4.7%)
BRA	2899 (53.0%)	1579 (28.9%)	794 (14.5%)	199 (3.6%)
RUS	3647 (67.0%)	763 (14.0%)	868 (15.9%)	165 (3.0%)

Table S3. The number of respondents who prefer World-AI for each diagnostic target (%).

		Diagnostic target					
		Myself	Family	Children	Significant Other	Friends	Stranger
Total	JPN1	1130 (22.6%)	960 (19.2%)	900 (18.0%)	1015 (20.3%)	1351 (27.0%)	2151 (43.0%)
	JPN2	1079 (21.6%)	913 (18.3%)	849 (17.0%)	964 (19.3%)	1308 (26.2%)	2270 (45.4%)
	US	1265 (25.1%)	1406 (27.9%)	1244 (24.7%)	1340 (26.6%)	1520 (30.2%)	2088 (41.5%)
	UK	1655 (32.9%)	1575 (31.3%)	1401 (27.8%)	1536 (30.5%)	1778 (35.3%)	2383 (47.3%)
	SWE	1811 (33.3%)	1655 (30.4%)	1525 (28.0%)	1663 (30.5%)	1918 (35.2%)	2579 (47.4%)
	TWN	772 (27.4%)	765 (27.1%)	751 (26.6%)	831 (29.5%)	946 (33.5%)	1367 (48.5%)
	AUS	755 (27.7%)	760 (27.9%)	694 (25.5%)	726 (26.7%)	827 (30.4%)	1084 (39.8%)
	BRA	1778 (32.5%)	1804 (33.0%)	1598 (29.2%)	1761 (32.2%)	1975 (36.1%)	2373 (43.4%)
	RUS	928 (17.0%)	871 (16.0%)	823 (15.1%)	925 (17.0%)	1167 (21.4%)	1631 (30.0%)
Male	JPN1	607 (24.3%)	534 (21.4%)	505 (20.2%)	519 (20.8%)	724 (29.0%)	1119 (44.8%)
	JPN2	594 (23.8%)	527 (21.1%)	493 (19.7%)	515 (20.6%)	740 (29.6%)	1218 (48.7%)
	US	679 (27.0%)	756 (30.0%)	670 (26.6%)	708 (28.1%)	845 (33.5%)	1138 (45.2%)
	UK	867 (34.4%)	837 (33.3%)	745 (29.6%)	806 (32.0%)	947 (37.6%)	1238 (49.2%)
	SWE	944 (34.9%)	851 (31.4%)	815 (30.1%)	843 (31.1%)	1017 (37.6%)	1353 (50.0%)
	TWN	433 (30.8%)	422 (30.0%)	417 (29.7%)	459 (32.7%)	539 (38.4%)	757 (53.9%)
	AUS	446 (33.0%)	430 (31.8%)	413 (30.5%)	424 (31.3%)	472 (34.9%)	625 (46.2%)
	BRA	941 (34.5%)	966 (35.4%)	860 (31.5%)	922 (33.8%)	1069 (39.2%)	1278 (46.9%)
	RUS	530 (19.5%)	507 (18.6%)	487 (17.9%)	523 (19.2%)	692 (25.4%)	926 (34.0%)
Female	JPN1	523 (20.9%)	426 (17.0%)	395 (15.8%)	496 (19.8%)	627 (25.1%)	1032 (41.3%)
	JPN2	485 (19.4%)	386 (15.4%)	356 (14.2%)	449 (18.0%)	568 (22.7%)	1052 (42.1%)

	US	586 (23.3%)	650 (25.8%)	574 (22.8%)	632 (25.1%)	675 (26.8%)	950 (37.7%)
	UK	788 (31.3%)	738 (29.3%)	656 (26.0%)	730 (29.0%)	831 (33.0%)	1145 (45.4%)
	SWE	867 (31.7%)	804 (29.4%)	710 (25.9%)	820 (29.9%)	901 (32.9%)	1226 (44.8%)
	TWN	339 (24.0%)	343 (24.2%)	334 (23.6%)	372 (26.3%)	407 (28.8%)	610 (43.1%)
	AUS	309 (22.6%)	330 (24.1%)	281 (20.5%)	302 (22.0%)	355 (25.9%)	459 (33.5%)
	BRA	837 (30.5%)	838 (30.5%)	738 (26.9%)	839 (30.6%)	906 (33.0%)	1095 (39.9%)
	RUS	398 (14.6%)	364 (13.4%)	336 (12.3%)	402 (14.8%)	475 (17.5%)	705 (25.9%)

Table S4. The differences in World-AI acceptance between diagnoses for oneself and for other targets. '+' (and '–') indicates a diagnostic target that had a larger (smaller) percentage of respondents who chose an Individual-AI diagnosis for themselves. Significance codes are as follows: '*' as $p < 0.05$, '**' as $p < 0.01$, '***' as $p < 0.001$, and 'n.s.' for insignificance performing Pearson's chi-square test with Yates' continuity correction.

		Diagnosis for myself vs for				
		Family	Children	Significant Other	Friend	Stranger
Total	JPN1	+ ***	+ ***	+ **	– ***	– ***
	JPN2	+ ***	+ ***	+ **	– ***	– ***
	US	– **	+	–	– ***	– ***
	UK	+	+ ***	+ *	– *	– ***
	SWE	+ **	+ ***	+ **	– *	– ***
	TWN	+	+	–	– ***	– ***
	AUS	–	+	+	– *	– ***
	BRA	–	+ ***	+	– ***	– ***
	RUS	+	+ **	+	– ***	– ***
Male	JPN1	+ *	+ ***	+ **	– ***	– ***
	JPN2	+ *	+ ***	+ **	– ***	– ***
	US	– *	+	–	– ***	– ***
	UK	+	+ ***	+	– *	– ***
	SWE	+ **	+ ***	+ **	– *	– ***
	TWN	+	+	–	– ***	– ***
	AUS	+	+	+	–	– ***
	BRA	–	+ *	+	– ***	– ***
	RUS	+	+	+	– ***	– ***
Female	JPN1	+ ***	+ ***	+	– ***	– ***
	JPN2	+ ***	+ ***	+	– **	– ***
	US	– *	+	–	– **	– ***
	UK	+	+ ***	+	–	– ***
	SWE	+	+ ***	+	–	– ***
	TWN	–	+	–	– **	– ***
	AUS	–	+	+	– *	– ***
	BRA	–	+ **	–	– *	– ***
	RUS	+	+ *	–	– **	– ***

Table S5. The independent chi-square test for each response between JPN1 (before the COVID-19 pandemic) vs. JPN2 (during the COVID-19 pandemic). '+' (and '–') indicates the percentage of respondents who chose a World-AI diagnosis in JPN1 that is larger (smaller) than that in JPN2. Significance codes are as follows: '*' as $p < 0.05$, '**' as $p < 0.01$, '***' as $p < 0.001$, and 'n.s.' for insignificance performing Pearson's chi-square test with Yates' continuity correction.

		Diagnosis target					
		Myself	Family	Children	Significant Other	Friends	Stranger
	Total	+	+	+	+	+	– *
Gender	Male	+	+	+	+	–	– **
	Female	+	+	+	+	+	–
Age	20s	–	–	–	–	–	–
	30s	–	+	–	+	+	–
	40s	+	+	+	+	+ *	–
	50s	+	+	+	+	+	–
	60s	+	+	+	+	–	– *

Dataset (separate file).

All the data related to this study are available at (doi:10.5061/dryad.nk98sf7wb):

Data_S1.csv: Responses collected at the first survey in Japan (JPN1)
Data_S2.csv: Responses collected at the second survey in Japan (JPN2)
Data_S3.csv: Responses collected at the survey in the United States of America (US)
Data_S4.csv: Responses collected at the survey in the United Kingdom (UK)
Data_S5.csv: Responses collected at the survey in Sweden (SWE)
Data_S6.csv: Responses collected at the survey in Taiwan (TWN)
Data_S7.csv: Responses collected at the survey in Australia (AUS)
Data_S8.csv: Responses collected at the survey in Brazil (BRA)
Data_S9.csv: Responses collected at the survey in Russia (RUS).
